# *Nocardia pseudobrasiliensis* Co-infection in SARS-CoV-2 Patients

**DOI:** 10.3201/eid2904.221439

**Published:** 2023-04

**Authors:** Daniel Beau Stamos, Aldo Barajas-Ochoa, Jillian E. Raybould

**Affiliations:** Virginia Commonwealth University Health System, Richmond, Virginia, USA

**Keywords:** nocardiosis, co-infection, *Nocardia pseudobrasiliensis*, SARS-CoV-2, COVID-19, opportunistic infections, bacteria, respiratory infections, coronavirus disease, severe acute respiratory syndrome coronavirus 2, United States, *Suggested citation for this article*: Stamos DB, Barajas-Ochoa A, Raybould JE. *Nocardia pseudobrasiliensis* co-infection in SARS-CoV-2 patients. Emerg Infect Dis. 2023 Apr [*date cited*]. https://doi.org/10.3201/eid2904.221439

## Abstract

During the SARS-CoV-2 pandemic, few cases of *Nocar­dia* spp. co-infection have been reported during or after a COVID-19 infection. *Nocardia* spp. are gram-positive aerobic actinomycetes that stain partially acid-fast, can infect immunocompromised patients, and may cause dis­seminated disease. We report the case of a 52-year-old immunocompromised man who had *Nocardia pseudobrasiliensis* pneumonia develop after a SARS-CoV-2 in­fection. We also summarize the literature for no­cardiosis and SARS-CoV-2 co-infections. *Nocardia* spp. infection should remain a part of the differential diagnosis for pneumonia in immunocompromised hosts, regardless of other co-infections. Sulfonamide/carbapenem combina­tions are used as empiric therapy for nocardiosis; species identification and susceptibility testing are required to se­lect the optimal treatment for each patient.

## Abstract 2

*Nocardia* spp. infection should remain on the differential diagnosis of pneumonia in immunocompromised hosts, regardless of co-infections.

*Nocardia pseudobrasiliensis* are gram-positive aerobic actinomycetes; stains of *N. pseudobrasiliensis* are partially acid fast (*1*). As with other *Nocardia* species, *N. pseudobrasiliensis* can infect immunocompromised patients and may cause disseminated disease (*2*). Risk factors for nocardiosis include immunosuppression caused by solid organ or hematopoietic cell transplantation, glucocorticoid therapy, chronic lung disease, diabetes, AIDS, and malignancy (*3*,*4*). Infection by other pathogens during or after SARS-CoV-2 infection is a known but relatively uncommon occurrence. Nocardiosis co-infection with SARS-CoV-2 is rarely reported. We describe a case of SARS-CoV-2 co-infection with pulmonary *Nocardia pseudobrasiliensis* and summarize the literature on nocardiosis and COVID-19 co-infection.

## Case Report

A 52-year-old man sought care at the emergency department at Virginia Commonwealth University Health System (Richmond, Virginia, USA) in July 2022 because of increased work of breathing after a positive home COVID-19 test. The patient’s symptoms began 10 days before admission. He reported that he had not experienced fevers, chills, sore throat, abdominal pain, or diarrhea. He was admitted for the management of hypoxia caused by COVID-19 pneumonia. His medical history included type 2 diabetes mellitus treated with empagliflozin, bronchiectasis, and multisystem sarcoidosis. His sarcoidosis was first diagnosed in 2011 by lung biopsy and had progressed to stage IV pulmonary sarcoidosis by 2019. An implantable cardioverter defibrillator was placed in 2019 to address cardiac sarcoidosis. For his sarcoidosis, the patient received oral hydroxychloroquine (200 mg 2×/d) and intravenous (IV) infliximab (800 mg every 8 wk). He was a former smoker who stopped 30 years earlier; he had a job as an apartment maintenance worker.

At the time of admission, the patient was hemodynamically stable and afebrile. Pertinent physical examination findings included increased work of breathing and wheezing. Cardiac and pulmonary examination were otherwise unremarkable. Chest radiograph showed findings related to sarcoidosis without acute cardiopulmonary disease. On hospital day 1, he started COVID-19 treatment with intravenous remdesivir (200 mg/d) and dexamethasone (6 mg/d). However, his hypoxia progressed; by hospitalization day 2, he required 5 L/min of oxygen delivered by face mask. Remdesivir was discontinued because of gastrointestinal side effects. Laboratory testing revealed a leukocyte count of 10.1 × 10^9^ cells/L, a C-reactive protein concentration of 15.0 mg/dL, and a positive COVID-19 PCR test.

**Figure 1:**
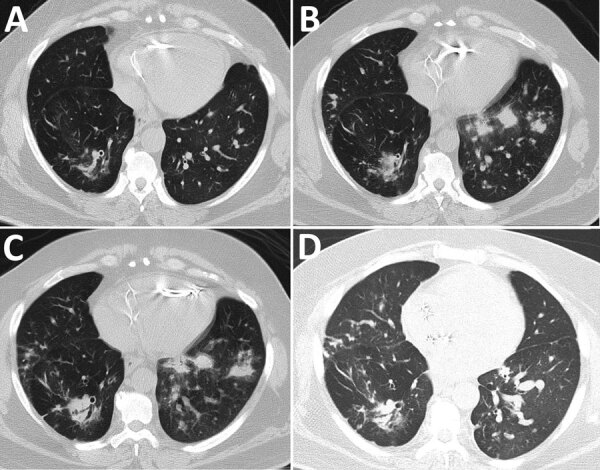
Computed tomography of the chest without contrast showing progression of illness in a 52-year-old immunocompromised man who experienced *Nocardia pseudobrasiliensis* pneumonia after a SARS-CoV-2 infection. A) Scan obtained 4 months before patient sought care. B) Scan on hospitalization day 5 shows development of lung nodules without ground-glass opacities in both lungs. C) Scan on hospitalization day 12 shows progression of nodules on the left lung. D) Scan obtained 28 days post-discharge shows improvement of pulmonary nodules.

Because the patient’s hypoxia continued to worsen, we obtained a chest computed tomography scan without IV contrast on hospital day 5. Imaging revealed progressive consolidative opacities bilaterally, most pronounced in the lung bases without a ground glass appearance (Figure 1, panel B). We obtained sputum cultures and started the patient on intravenous piperacillin/tazobactam (3.375 g every 6 h) to treat suspected bacterial pneumonia.

**Figure 2:**
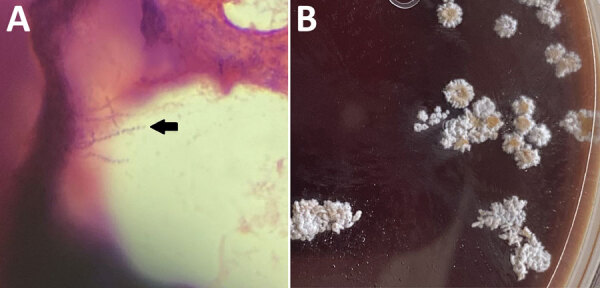
Histopathology of samples from a 52-year-old immunocompromised man who experienced *Nocardia pseudobrasiliensis* pneumonia after a SARS-CoV-2 infection. A) Branching gram-positive rods (arrow) seen in Gram stain of *Nocardia* culture plate. Original magnification ×100. B) *N. pseudobrasiliensis* colonies seen on a *Nocardia* culture plate with characteristic chalky white and orange pigmentation.

On hospital day 7, sputum Gram stain revealed beaded, gram-positive rods concerning for *Nocardia* spp. (Figure 2, panel A). The patient’s antimicrobial treatments were transitioned to intravenous trimethoprim/sulfamethoxazole (TMP/SMX) (5 mg/kg every 8 h) and imipenem (500 mg every 6 h) pending confirmation of suspected *Nocardia* spp. Despite dual therapy, the patient’s hypoxia persisted; a repeat chest CT without IV contrast on hospital day 12 showed progression of a consolidative nodular opacity in the left lung base (Figure 1, panel C). Because the patient was not responding to treatment, we added oral linezolid (600 mg every 12 h) to his antimicrobial regimen.

After initiation of linezolid with continued imipenem and TMP/SMX therapy, the patient demonstrated gradual improvement; his hypoxia eventually resolved. On hospital day 16, species identification with matrix-assisted laser desorption/ionization time-of-flight mass spectrometry confirmed *N. pseudobrasiliensis.* Colonies from the *Nocardia* culture plate had the characteristic chalky white appearance and orange pigmentation (Figure 2, panel B). The susceptibility profile (conducted using broth dilution by LabCorp) demonstrated imipenem resistance (Table 1). Because the resistance to imipenem and hyperkalemia was likely caused by TMP/SMX, the patient was discharged on hospital day 20 on linezolid (600 mg 2×/d) and ciprofloxacin (500 mg 2×/d) with plans to continue dual therapy for >6 weeks after discharge. Of note, a magnetic resonance image of the brain with contrast, which we performed before discharge, did not show ring-enhancing lesions or signs of central nervous system nocardiosis. At the 2-week follow-up visit, the patient’s shortness of breath improved and he no longer needed supplemental oxygen at rest but did intermittently require supplemental oxygen on exertion. Imaging at 28 days after discharge showed improvement of the pulmonary nodules (Figure 1, panel D). After a month of outpatient treatment, the patient’s symptoms continued to improve, but he experienced a break in therapy for 2 weeks because of financial constraints, after which the linezolid was switched to TMP/SMX (2 double-strength tablets of 160 mg TMP and 800 SMX every 8 h) and oral ciprofloxacin (500 mg 2×/d). After discussion with the patient, we decided to continue dual antimicrobial therapy because of his need to continue taking glucocorticoids for his sarcoidosis. At follow-up 4 months after discharge, the patient reported dyspnea only with heavy exertion and no medication side effects. He would undergo repeat imaging of the chest with plans to transition to TMP/SMX monotherapy if the imaging shows significant improvement of the pulmonary nodules.

**Table 1:** Reported susceptibility of *Nocardia pseudobrasiliensis* bacteria to antimicrobial drugs*

Antimicrobial drug	This study	Case 1†	Case 2†	Case 3†	Case 4†	Case 5†	Case 6†	Case 7‡
Amikacin	S	R	S	II	S	S	S	VSR or II
Amoxi-clav	R	R	II	S	R	R	R	R
Ceftriaxone	I	R	R	II	S	S	R	VSR or II
Ciprofloxacin	S	S	S	S	S	S	S	II
Clarithromycin	S	S	II	S	S	S	S	S
Doxycycline	R	R	II	II	II	R	R	II
Imipenem	R	R	R	R	II	R	R	VSR or II
Linezolid	S	S	S	II	II	S	S	S
Minocycline	R	R	II	II	M	R	R	II
Moxifloxacin	S	S	II	II	II	S	II	II
Tobramycin	S	II	II	II	II	II	II	II
TMP/SMX	S	R	R	II	S§	R	S	S

*Amoxi-clav, amoxicilline/clavulanic acid; I, intermediate; II, insufficient information; M, moderate; R, resistant; S, susceptible; TMP/SMX, trimethoprim/sulfamethoxazole; VSR, variable susceptibility results. †Veerappan Kandasamy et al. (*2*). ‡Wilson et al. (*3*). §This specimen was susceptible to SMX only.

## Discussion

This patient had a previous COVID-19 infection and multiple other risk factors for pulmonary nocardiosis, including diabetes mellitus, bronchiectasis, and immunosuppression caused by sarcoidosis treatment. To date, 10 cases of nocardiosis during or shortly after COVID-19 infection have been reported (Table 2). All patients had risk factors that predisposed them to infection with *Nocardia* spp. (*2*–*4*). Most patients experienced nocardiosis 5–50 days after their SARS-CoV-2 diagnosis; average time to co-infection identification was 17 days. One immunosuppressed patient experienced brain nocardiosis 200 days after their initial COVID-19 diagnosis but had persistently tested positive by reverse transcription PCR for SARS-CoV-2 during that time. Of note, all patients received glucocorticoids during their hospitalizations. 

**Table 2:** Nocardiosis infections reported during or shortly after COVID-19 infection, 2021*

Study	Age, y/sex	*Nocardia* species; positive culture site(s)	Days from SARS-CoV-2 diagnosis to *Nocardia* diagnosis	Site(s) of *Nocardia* infection	Predisposing factor	On MV?	Steroids before *Nocardia* diagnosis	Susceptibilities	Final therapy	Outcome
Colaneri et al. (*5*)	45/F	*N. cryarcigeorgica*; subcutaneous lumps, lower respiratory tract sputum smear and culture	5 d	Lung, skin, kidney	AIDS	No	Hydrocortisone for persistent fever	Susceptible: TMP/SMX, amikacin, linezolid; resistant: AMO/CLA, third-generation cephalosporins, quinolones	Tedizolid for 1 wk followed by 1 y TMP/SMX	Survived
Arif et al. (*6*)	61/F	*N. farcinica*; Gram stain of pulmonary nodule biopsy	10 d	Lung	Type 2 diabetes mellitus	No	Dexamethasone for COVID-19	NA	TMP/SMX and linezolid	Survived
Atemnkeng et al. (*7*)	63/M	*N. asteroids;* bronchial lavage culture	50 d	Lung, brain	Type 2 diabetes mellitus	No	Dexamethasone for COVID-19	Resistant: carbapenem	TMP/SMX and linezolid for 1 y	Survived
Kaur and Bhatti (*8*)	80/F	*N. farcinica;* Gram stain and culture of brain abscess biopsy	>10 d, exact date unspecified	Brain	Advanced age	No	Dexamethasone for COVID-19	NA	TMP/SMX, ceftriaxone, doxycycline	Not reported
Driscoll et al. 2022 (*9*)	16/M	*N. farcinica;* lower airway samples	9 d	Lung	Cystic fibrosis, bronchiectasis	Yes	Dexamethasone for COVID-19	NA	Linezolid	Died
Cicero et. al. 2022 (*10*)	79/M	*N. otitidiscaviarum:* sputum culture	17 d	Lung	COPD, previous pulmonary tuberculosis, cirrhosis	No	Methylpredisolone for COVID-19	Susceptible: ceftriaxone, amikacin, ciprofloxacin, meropenem, TMP/SMX	Not given	Died
Velickovic et al. 2022 (*11*)	30/F	*N. cyriacigeorgica*; brain abscess	200 d	Brain	Systemic lupus erythematosus, glucocorticoid therapy, CD4 count <100	No	Prednisone for systemic lupus erythematosus	Susceptible: TMP/SMX, ceftriaxone, cefotaxime, imipenem, linezolid; resistant: ampicillin, AMO/CLA, fluoroquinolones	TMP/SMX	Survived
DiMeglio et al. 2022 (*12*)	70/M	*N. farcinica:* spinal cord abscess	48 d	Brain and spinal cord	Diabetes mellitus, advanced age, glucocorticoid therapy	No	Dexamethasone for COVID-19	NR	TMP/SMX, linezolid	Died
Laplace et al. 2022 (*13*)	83/M	*N. cyriacigeorgica:* sputum culture	4 d	Lung	Advanced age	No	Dexamethasone for COVID-19	NR	Imipenem, cotrimoxazole	Died
This study	52/M	*N. pseudobrasiliensi*s: sputum gram stain	7 d	Lung	Bronchiectasis, sarcoidosis, immunosuppressive therapy, type 2 diabetes mellitus	No	Dexamethasone for COVID-19	Susceptible: amikacin, ciprofloxacin, clarithromycin, linezolid, moxifloxacin, tobramycin, TMP/SMX; resistant: AMO/CLA, doxycycline, imipenem, minocycline	Linezolid, ciprofloxacin	Survived

*AMO/CLA, amoxicillin/clavulanic acid; COPD, chronic obstructive pulmonary disease; MV, mechanical ventilation; NA, not available; NR, not reported; TMP/SMX, trimethoprim/sulfamethoxazole.

Pulmonary nocardiosis was the most common site of infection, occurring in 7 patients. Central nervous system nocardiosis occurred in 4 patients. Five different species of *Nocardia* were identified, but *N. farcinica* was the most frequently isolated species. This heterogeneity likely explains why each patient was ultimately discharged on a different antimicrobial regimen (Table 2); *Nocardia* species have unique susceptibilities (*2*,*3*,*14*). Our patient’s delayed clinical improvement until the addition of linezolid highlights the clinical importance of *Nocardia* species identification and susceptibilities. Ciprofloxacin, clarithromycin, and linezolid are typically effective against *N. pseudobrasiliensis*, whereas TMP/SMX susceptibility varies between cases (Table 1).

The overall risk for all-cause co-infection in patients with COVID-19 appears to be low. A cohort study by Garcia-Vidal et al. (*15*) analyzed 989 patients admitted to the hospital with COVID-19 and found that only 31 patients had a community-acquired co-infection at the time of COVID-19 diagnosis; 25/31 patients had bacterial co-infections. Garcia-Vidal et al. also observed 51 total cases of hospital-acquired co-infections diagnosed in 43 patients; 44/51 cases were bacterial co-infections. However, when focusing on COVID-19 patients requiring invasive mechanical ventilation, the incidence of co-infection appears to be higher. Søvik et al. (*16*) reviewed 156 patients who required mechanical ventilation while infected with COVID-19 and evaluated those patients for co-infection. A total of 67 patients experienced 90 co-infections, 78% of which involved the lower airways; no *Nocardia* spp. infections were reported. Co-infection was strongly associated with dexamethasone use, underlying autoimmune disease, and length of intensive care stay (*16*). Despite these findings, mechanical ventilation was unlikely to be an independent predisposing factor for pulmonary nocardiosis; 1 patient required mechanical ventilation during their hospitalization.

Analytical epidemiologic studies are needed to assess whether SARS-CoV-2 infection is an independent risk factor for nocardiosis. However, mechanical and immune mechanisms after COVID-19 infection may play a role in *Nocardia* spp. co-infection. Paget and Trottein (*17*) recently described how influenza virus infection can cause direct or indirect damage to the respiratory barrier, creating conditions for bacterial attachment and translocation, and lead to macrophage, neutrophil, and natural killer cell dysfunction which result in poor bacterial control. COVID-19 infection might promote co-infections by opportunistic pathogens such as *Nocardia* spp. although these mechanisms are not well studied. Because nocardiosis is rarely transmitted in the nosocomial setting (*4*), the patients identified in this series were likely colonized with *Nocardia* spp. before admission; all of the patients had factors that made them immunocompromised and received steroids before receiving their *Nocardia* diagnosis (Table 2). It is possible that a COVID-19 infection and the glucocorticoid therapy used to treat it synergistically trigger *Nocardia* spp. co-infection in patients who are already chronically immunocompromised because of the combination of additional immunosuppression and permissive parenchymal conditions. Although a synergistic relationship between COVID-19 and glucocorticoid use is plausible, the data in this study are observational; analytical studies would further clarify the association.

In summary, *Nocardia* bacteria can be a cause of co-infection in patients with COVID-19 pneumonia that may present as further respiratory deterioration. However, *Nocardia* spp. has not been reported in reviews as a cause of co-infection in patients with COVID-19 pneumonia (*16*,*18*,*19*). Immunocompromised patients, such as those on glucocorticoid therapy, those who have received solid organ or hematopoietic cell transplantation, and those positive for HIV, are at higher risk for nocardiosis. Clinicians should include nocardiosis in the differential diagnosis for immunosuppressed patients with severe pneumonia and assess for disseminated disease and central nervous system involvement, especially in the context of potent steroid use to treat immunocompromised patients with COVID-19. Sulfonamide–carbapenem combinations are used as empiric therapy for nocardiosis, but species identification and susceptibility testing are required to select optimal treatment.
